# Target sequencing of 307 deafness genes identifies candidate genes implicated in microtia

**DOI:** 10.18632/oncotarget.18803

**Published:** 2017-06-28

**Authors:** Pu Wang, Xinmiao Fan, Yibei Wang, Yue Fan, Yaping Liu, Shuyang Zhang, Xiaowei Chen

**Affiliations:** ^1^ Department of Otolaryngology, Peking Union Medical College Hospital, Peking Union Medical College and Chinese Academy of Medical Sciences, Beijing, China; ^2^ Department of Medical Genetics, School of Basic Medicine, Peking Union Medical College, Peking Union Medical College and Chinese Academy of Medical Sciences, Beijing, China; ^3^ Department of Cardiology, Peking Union Medical College Hospital, Peking Union Medical College and Chinese Academy of Medical Sciences, Beijing, China

**Keywords:** microtia, deafness genes, next-generation sequencing, SKAT

## Abstract

Microtia is a congenital malformation of the external ear caused by genetic and/or environmental factors. However, no causal genetic mutations have been identified in isolated microtia patients. In this study, we utilized targeted genomic capturing combined with next-generation sequencing to screen for mutations in 307 deafness genes in 32 microtia patients. Forty-two rare heterozygous mutations in 25 genes, including 22 novel mutations in 24 isolated unilateral microtia cases were identified. Pathway analysis found five pathways especially focal adhesion pathway and ECM-receptor interaction pathway were significantly associated with microtia. The low-frequency variants association study was used and highlighted several strong candidate genes *MUC4, MUC6, COL4A4, MYO7A, AKAP12, COL11A1, DSPP, ESPN, GPR98, PCDH15, BSN, CACNA1D, TPRN*, and *USH1C* for microtia (*P* = 2.51 × 10^−4^). Among these genes, *COL4A4* and *COL11A1* may lead to microtia through focal adhesion pathway and ECM-receptor interaction pathway which are connected to the downstream *Wnt* signaling pathway. The present results indicate that certain genes may affect both external/middle and inner ear development, and demonstrate the benefits of using a capture array in microtia patients.

## INTRODUCTION

Microtia is a congenital developmental malformation of the external ear that ranges in severity from mild differences in auricular shape and size to complete absence of the external ear with atresia or stenosis of the auditory canal [1,2]. Prevalence rates of this uncommon anomaly have been reported to vary from 0.83 to 4.34 per 10,000 [3]. It can occur unilaterally (79%-93%) and bilaterally [2–4], and approximately 60% of unilateral microtia are seen on the right side [5,6]. Microtia is divided into four grades, according to the Marx classification system, widely used to describe the degree of microtia. In grade I microtia, all of the outer ear structures are normal, but the pinna is small. This can occur with or without aural atresia. In grade II, auricle is abnormal, but definable. Grade III displays only soft tissue rudiments, with no definable structures of the auricle. Grade IV is the extreme case in which the external ear and auditory canal are absent, a condition called anotia [7].

Microtia can occur as an isolated birth defect or as part of a spectrum of anomalies or a syndrome. The most common associated malformations are cleft lip and/or palate, limb reduction defects, renal anomalies, vertebral abnormalities, and cardiac defects [3,4,8]. Although differences in the development of the external/middle ear and inner ear during embryogenesis mean that external and middle ear malformations can occur without inner ear malformations and vice versa, malformations in microtia can affect the external ear, middle ear, and/or inner ear in combination [3,4,8]. Swartz and Faerber reported inner ear malformation frequencies of 11% to 30% in individuals with outer and middle ear malformations [9].

In addition, many syndromic microtia patients present with inner ear malformations, including branchio-oculofacial, branchio-oto-renal/branchio-otic, CHARGE (coloboma, heart defects, choanal atresia, retarded growth and development, genital abnormalities, ear anomalies), Nager (preaxial acrofacial dysostosis), Treacher Collins, Wildervanck (cervico-oculo-acoustic), LAMM (congenital deafness, inner ear agenesis, microtia, microdontia) and lacrimo-auriculodento-digital (LADD) syndromes, among others. The frequent malformations of the inner ear are common cavity, cochlear aplasia, Mondini defect, enlarged vestibular aqueduct, abnormal/absent semicircular canals, and bulbous IAC (internal auditory canal) [7]. Conductive hearing loss, sensorineural hearing loss, and mixed hearing loss can all be found in microtia patients [7]. Given these observations, we propose that malformations of the external/middle ear and inner ear may reflect the actions of certain genes in common.

To date, no studies have reported screens of deafness genes in microtia patients. The technology of targeted genomic capturing combined with next-generation sequencing (NGS) has proven to be effective and feasible and has been successfully used in several studies [10,11]. Here, we utilized this approach to screen for mutations in 307 targeted genes in 32 isolated unilateral microtia patients using a customized capture array that includes all known deafness genes, selected from human and animal experimental studies (Supplementary Table 1). We attempted to identify mutations or genes that may be associated with isolated unilateral microtia and the signaling pathways that may be responsible for this disease.

## RESULTS

### Variant identification

A total of 4899 exons comprising 2,362,343 bases (1,441,253 in target regions and 921,090 in flanking regions) were captured and sequenced in our study. After alignment to the reference human genome (NCBI37/hg19), 70.93% of the clean reads could be matched to the targeted regions. The average read depth for the targeted regions was 394.5-fold, and 97.68% of the targeted regions were covered by 20 or more reads, demonstrating the high quality of the sequencing. A detailed coverage analysis for each sample is provided in Supplementary Figure 1.

### Identification of rare or novel mutations

Using a rigorous filtering pipeline, we identified 42 very rare heterozygous mutations, including 22 novel mutations in 25 genes from 24 isolated unilateral microtia patients (Table 1). A breakdown of the 42 mutations is as follows: 36 missense mutations, 3 frameshift variants, 2 splice mutations and 1 nonsense mutation. Twenty-one of the 36 missense mutations were predicted to be pathogenic by the Condel program. The three frameshift variants were c.2081_2082insC (p.P695Tfs*71) in *DIAPH1* (diaphanous-related formin 1), c.3300_3301delAA (p.K1103Afs*16) in *ALMS1* (Alstrom syndrome 1), and c.6072delG (p.R2025Afs*6) in *OTOG* (otogelin). One mutation, c.4248C>A in *MYO7A* (myosin VIIA), was predicted to lead to a truncated protein owing to a premature stop codon. Mutations in *ALMS1, COL4A4* (collagen type IV alpha 4 chain), *GPR98* (G protein-coupled receptor 98), *MYO15A, MYO7A*, and *USH1C* (Usher syndrome 1C) genes occurred in two or more cases (Allele frequency ≥ 1.56%) (Table 2).

**Table 1:** Mutations identified in isolated unilateral microtia patients

Sample ID	Gene Symbol	Type of variation	Mutation	PhyloP in Vertebrates	Condel Score	Condel Prediction	Novel
**X242**	*ESPN*	missense	c.2171C>T, p.Pro724Leu	1.116	0.836	deleterious	Reported
	*GPR98*	missense	c.11059C>T, p.Arg3687Cys	3.779	0.69	deleterious	Novel
	*DIAPH1*	frameshift	c.2081_2082insC, p.Pro695Thrfs*71	.	.	.	Reported
	*MYO15A*	missense	c.6559C>T, p.Arg2187Cys	1.942	.	.	Reported
**X243**	*FGFR2*	missense	c.1388T>A, p.Val463Asp	4.509	0.718	deleterious	Reported
	*MYO7A*	missense	c.5421G>T, p.Glu1807Asp	0.519	0.74	deleterious	Novel
**X245**	*HOXA1*	missense	c.344A>G, p.Gln115Arg	2.769	.	.	Novel
**X246**	*COL9A1*	missense	c.2636C>A, p.Pro879His	3.371	0.857	deleterious	Novel
**X248**	*MYO7A*	missense	c.3397G>A, p.Gly1133Arg	3.778	.	.	Novel
	*MYO7A*	nonsense	c.4248C>A, p.Tyr1416*	2.927	.	.	Reported
**X250**	*COL4A4*	missense	c.3849T>A, p.Ser1283Arg	−0.336	.	.	. Novel
**X251**	*DSPP*	missense	c.2615A>C, p.Asp872Ala	1.275	.	.	Reported
**X252**	*TRIOBP*	missense	c.2777G>A, p.Arg926His	2.956	0.786	deleterious	Reported
**X254**	*ALMS1*	missense	c.162G>C, p.Glu54Asp	0.429	.	.	. Novel
	*USH1C*	missense	c.2329G>A, p.Gly777Arg	5.095	0.945	deleterious	Reported
	*MYO1A*	splice-5	c.1164+1delG	5.634	.	.	Novel
**X256**	*MYO15A*	missense	c.7100G>T, p.Gly2367Val	0.015	.	.	Novel
**X260**	*COL4A4*	missense	c.1537A>T, p.Ser513Cys	0.06	0.481	deleterious	Novel
**X262**	*PCDH15*	missense	c.5213C>T, p.Pro1738Leu	0.98	.	.	Novel
**X263**	*GPSM2*	missense	c.122G>A, p.Arg41His	6.144	0.667	deleterious	Reported
**X265**	*ALMS1*	missense	c.8638T>C, p.Cys2880Arg	0.347	.	.	Reported
**X267**	*COL4A3*	missense	c.1103G>A, p.Arg368His	0.727	0.74	deleterious	Reported
**X267**	*USH1C*	missense	c.1275G>C, p.Lys425Asn	1.686	0.779	deleterious	Reported
**X270**	*OTOG*	missense	c.8129T>A, p.Val2710Glu	3.867	0.704	deleterious	Novel
**X274**	*WFS1*	missense	c.292G>A, p.Gly98Arg	4.158	0.945	deleterious	Novel
**X274**	*DIAPH3*	missense	c.772T>C, p.Tyr258His	3.193	0.8	deleterious	Novel
**X275**	*GPR98*	missense	c.13356G>C, p.Leu4452Phe	0.082	0.75	deleterious	Novel
	*ESPN*	missense	c.2171C>T, p.Pro724Leu	1.116	0.836	deleterious	Reported
**X244**	*ALMS1*	frameshift	c.3300_3301delAA, p.Lys1103Alafs*16	.	.	.	Novel
**X249**	*BSND*	missense	c.919C>T, p.Leu307Phe	3.393	0.809	deleterious	Reported
	*ALMS1*	missense	c.7495G>C, p.Glu2499Gln	0.375	.	.	Novel
	*COL4A4*	missense	c.4673C>T, p.Ala1558Val	2.594	.	.	Reported
	*GPR98*	missense	c.5233C>T, p.Arg1745Cys	2.647	0.945	deleterious	Novel
	*LOXHD1*	missense	c.4459G>A, p.Asp1487Asn	5.879	0.849	deleterious	Novel
**X258**	*COL4A4*	missense	c.1129C>T, p.Arg377Cys	0.582	.	.	Reported
	*OTOG*	frameshift	c.6072delG, p.Arg2025Alafs*6	3.056	.	.	Novel
**X261**	*COL4A4*	missense	c.1006C>G, p.Leu336Val	−0.162	.	.	Reported
	*TPRN*	missense	c.1927G>A, p.Val643Met	0.363	0.676	deleterious	Novel
**X266**	*USH2A	splice-3	c.8559-2A>G	4.853	.	.	Novel
	*GPR98*	missense	c.6303G>C, p.Lys2101Asn	1.027	0.481	deleterious	Reported
	*PCDH15*	missense	c.2047C>T, p.Arg683Cys	4.357	0.799	deleterious	Reported
**X271**	*COL11A1*	missense	c.1289C>A, p.Pro430His	5.849	.	.	Reported
	*COL4A4*	missense	c.2840G>A, p.Arg947Gln	−4.674	.	.	Reported

*indicates the mutation has been reported in HGMD database

**Table 2:** Mutations identified in the same gene in two or more patients

Sample ID	X242	X243	X244	X248	X249	X250	X254	X256	X258	X260	X261	X265	X266	X267	X271	X275
***ALMS1***			*c.3300_3301delAA*		*c.7495G>C*		*c.162G>C*					*c.8638T>C*				
***COL4A4***					*c.4673C>T*	*c.3849T>A*			*c.1129C>T*	*c.1537A>T*	*c.1006C>G*				*c.2840G>A*	
***GPR98***	*c.11059C>T*				*c.5233C>T*								*c.6303G>C*			*c.13356G>C*
***MYO15A***	*c.6559C>T*							*c.7100G>T*								
***MYO7A***		*c.5421G>T*		*c.3397G>A; c.4248C>A*												
***USH1C***							*c.2329G>A*							*c.1275G>C*		

**Figure 1:**
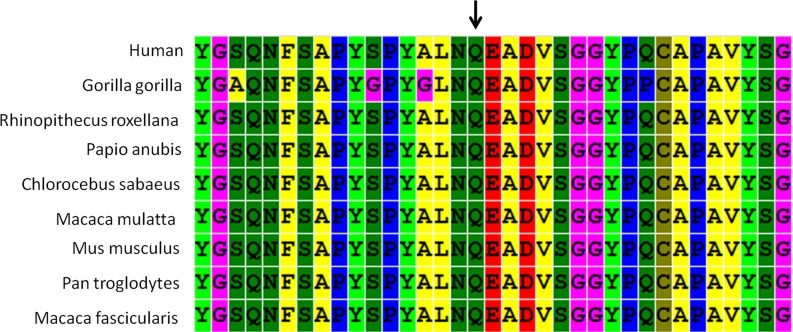
ConSeq conservation analysis of amino acid Q115 in the homeodomain of *HOXA1* This analysis is based on an alignment of nine full-length *HOXA1* proteins from different species. Different colors indicate different types of residues. The arrow shows the Q115 residue.

In addition, two intronic variants were identified; one, a c.8559-2A>G mutation in *USH2A*, has been reported to be pathogenic in HGMD, and the other, a c.1164+1delG mutation in *MYO1A*, was predicted to most likely affect splicing by the Human Splicing Finder program (http://www.umd.be/HSF3/). An alignment of a selected region of the *HOXA1* protein showed that amino acid 115 exhibited very high evolutionary conservation (Figure 1). A structural analysis of *HOXA1* protein performed using the HOPE server suggested that the mutation (c. 344 A>G, p. Q115R), which introduces a positively charged arginine for the negatively charged glutamine in the wild-type protein, could cause repulsion of ligands or other residues with the same charge.

### KEGG pathway enrichment analysis

**Figure 2:**
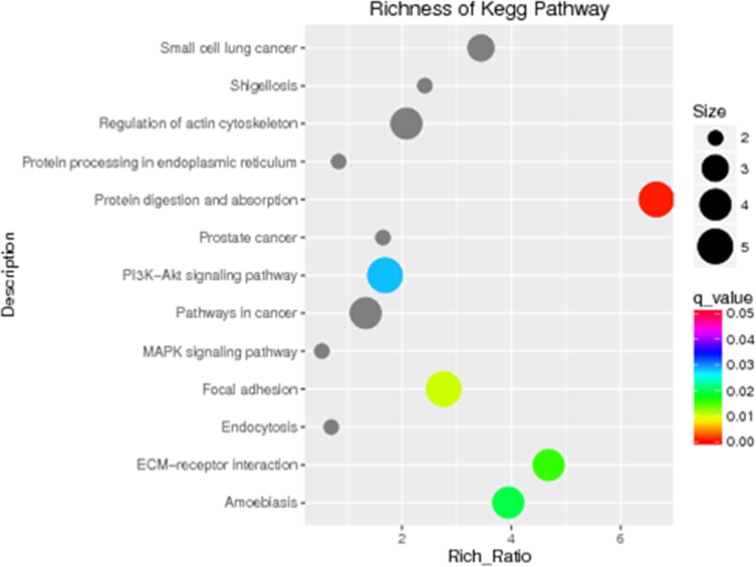
KEGG pathway analysis identified five signaling pathways that are significant in microtia (P < 0.05) KEGG pathway enrichments are displayed in a scatter diagram, where each point represents the enrichment level, the color corresponds to the q-value, and the size corresponds to the number of genes enriched for the given pathway. Red indicates the q-value; the smaller the value, the more significant the pathway enrichment.

To further determine the signaling pathways in which the 25 rare mutated genes found in microtia patients are involved, we performed a KEGG pathway enrichment analysis. This analysis revealed that five pathways are significantly associated with isolated unilateral microtia: the protein digestion and absorption pathway, the focal adhesion pathway, the extracellular matrix (ECM)-receptor interaction pathway, the amoebiasis pathway, and the phosphoinositide 3-kinase (PI3K)/Akt signaling pathway (Figure 2). Among these pathways, two (the focal adhesion pathway and ECM-receptor interaction pathway) may play important roles in auricular development, because both have been reported to be associated with cartilage destruction and the main component of the auricle is cartilage [12]. Notably, the two pathways are connected to the downstream *Wnt* signaling pathway. Based on these considerations, we propose that both pathways are linked to microtia (http://www.genome.jp/kegg/pathway.html).

### Association analysis

To test whether low-frequency mutations exist that might be risk factors for microtia, we conducted an association analysis of 32 isolated unilateral microtia cases and 208 healthy controls from China. After correction for multiple testing (*P* = 2.51 × 10^−4^), *MUC4* (mucin 4), *MUC6, COL4A4, MYO7A, AKAP12* (A-kinase anchoring protein 12), *COL11A1, DSPP* (dentin sialophosphoprotein), *ESPN* (espin), *GPR98, PCDH15* (protocadherin 15), *BSN* (bassoon), *CACNA1D* (calcium channel, voltage-dependent, L type, alpha 1D subunit), *TPRN* (taperin), and *USH1C* were identified as significantly associated with isolated unilateral microtia (Table 3, Supplementary Table 2). Of these genes, two were related to the pathways we identified above using KEGG pathway enrichment analysis. *COL4A4* was related to the pathways for ECM-receptor interaction and focal adhesion, and *COL11A1* was related to protein digestion and absorption (http://www.genecards.org/).

**Table 3:** Results of gene-based, low-frequency variant association tests (significance level *P* = 2.51 × 10-4)

Gene	Markers	*P*-value
***MUC4***	141	3.68 × 10^−17^
***MUC6***	95	1.20 × 10^−11^
***COL4A4***	9	2.24 × 10^−9^
***MYO7A***	8	2.02 × 10^−7^
***AKAP12***	6	1.82 × 10^−6^
***COL11A1***	7	1.82 × 10^−6^
***DSPP***	3	1.82 × 10^−6^
***ESPN***	5	1.82 × 10^−6^
***GPR98***	9	3.42 × 10^−6^
***PCDH15***	5	1.58 × 10^−5^
***BSN***	7	5.97 × 10^−5^
***CACNA1D***	4	0.000133
***TPRN***	4	0.000133
***USH1C***	4	0.000133

## DISCUSSION

During early development of vertebrates, signaling between neural crest cells and other embryonic cell types (e.g., endothelia and craniofacial ectoderm) plays a critical role in driving facial outgrowth and morphogenesis, including that of the external and inner ear [13]. The external ear begins its development during the fifth week, when hillocks developed from the first and second pharyngeal arches progress over several months to form the auricle. The otic placode, also derived from second pharyngeal arch begins to form on inner ear structures in the third week of embryonic development and is completed after several weeks [13]. Hypoplasia of the first and second pharyngeal arches is the underlying cause of microtia, a congenital malformation of the external ear. Thus, we propose that external and inner ear development may be regulated by certain genes in common. To date, no studies have reported an investigation of the associations between external ear deformities and deafness genes.

In the current study, we screened all exons of 307 deafness-associated genes, identifying 42 rare or novel mutations of 25 genes in isolated unilateral microtia patients. All of these mutations add to the database of potential pathogenic mutations in isolated unilateral microtia cases. Among these muations, only one, c.8559-2A>G in *USH2A*, has been reported to be pathogenic; when combined with p.T3936P or p.R34fs as a compound heterozygous mutation, it results in Usher syndrome type II [15]. This information could be helpful in providing genetic counseling for patients with related conditions.

The wingless/INT (Wnts) signaling pathway, together with bone morphogenetic proteins (BMPs) and fibroblast growth factors (FGFs), have been reported to be involved in external ear development [16]. Here, through pathway enrichment analysis of the 25 genes, we identified additional five pathways—protein digestion and absorption pathway, focal adhesion pathway, ECM-receptor interaction pathway, amoebiasis pathway and PI3K-Akt signaling pathway—that may be related to microtia. Of these pathways, two (focal adhesion pathway and ECM-receptor interaction pathway) may especially play more important roles during auricular development. Because both have been reported to be associated with cartilage destruction, and the main component of the auricle is cartilage [12]. More importantly, both pathways are connected to downstream Wnt signaling pathway, and thus we propose that they linked to microtia.

In addition to exploring rare mutations that may cause microtia, we also employed associated studies to identify potential pathogenic genes among low-frequency variants. The contribution of rare and low-frequency variants to human traits has been largely unexplored [17,18]. Using the candidate gene approach for complex traits, several previous rare variant association studies (RAVS) have successfully identified novel associations [19,20]. In the current study, through the low-frequency variants association studies by SKAT method, we identified several genes significantly associated with isolated unilateral microtia, including *MUC4, MUC6, COL4A4, MYO7A, AKAP12, COL11A1, DSPP, ESPN, GPR98, PCDH15, BSN, CACNA1D, TPRN*, and *USH1C*. Of these genes, two were related to the pathways we identified using KEGG pathway enrichment analysis. *COL4A4* was related to the pathways for ECM-receptor interaction and focal adhesion, and *COL11A1* was related to protein digestion and absorption. Thus we suggest that the two genes, *COL4A4* and *COL11A1*, may be strongly involved in the mechanism of microtia and are worth exploring in the context of the pathogenesis of microtia.

Additionally, a novel heterozygous mutation (c. 344A>G) in *HOXA1* identified in one isolated unilateral microtia patient also attracted our interest. It resulted in substitution of an arginine for a glutamine at position 115 (Q115R), which is highly evolutionarily conserved. A structural analysis of *HOXA1* protein performed using the HOPE server indicated that this mutation, which replaces a negatively charged residue in the wild-type with a positively charge amino acid, could cause repulsion of ligands or other residues with the same charge. *Hox* genes encode proteins that share a 60-amino-acid domain called the homeodomain. *HoxA* cluster genes play a fundamental role in building the sensorimotor circuitry in vertebrates. Mutations in either *HOXA1* or *HOXA2* have reported to result in external ear deformities, although the phenotypes caused by *HOXA1* mutations are more severe than those caused by *HOXA2* mutations. Diseases associated with *HOXA1* mutations include Athabaskan Brainstem Dysgenesis Syndrome and Bosley-Salih-Alorainy syndrome [21,22].

A small number of studies have also focused on the *HOXA2* gene as a genetic cause of isolated microtia. Identification of pathogenic mutations in *HOXA2* was reported in three different isolated bilateral microtia families [23–25], prompting researchers to propose that *HOXA2* may be among genes responsible for the pathogenesis of isolated microtia. However, studies that have focused on sporadic microtia patients have found no pathogenic mutations in *HOXA2* [26,27]. Consistent with this, we failed to identify any mutations in *HOXA2*, suggesting that there may be distinct etiologies that result in isolated microtia.

In conclusion, some deafness genes may be associated with microtia, and certain genes may affect both external/middle and inner ear development. Pathway analysis showed that two pathways—the focal adhesion and ECM-receptor interaction pathways—were associated with microtia. The deafness genes panel has the potential to enable the discovery of pathogenic genes in microtia patients and can be reliably used in such patients.

## MATERIALS AND METHODS

### Recruitment of subjects

Thirty-two isolated unilateral microtia patients (22 males, 10 females; mean age, 9 years; range, 6–28 years) were recruited from Peking Union Medical College Hospital (PUMCH). They were all classified as grade III external ear deformities, according to the classification by the Marx classification system [7], and had atresia of the auditory canal. None of the patients exhibited other complicating deformities. Patients with syndromic microtia such as Treacher Collins syndrome, Miller syndrome, CHARGE syndrome or branchio-oto-renal (BOR) syndrome, were excluded from the study. All patients were asked to provide a detailed family history, and none had a family history of microtia.

Ethical approval for this study was obtained from the institutional review board of PUMCH. All subjects or their parents gave written, informed consent to participate in this study.

### Targeted genomic capture and next-generation sequencing

Patients’ genomic DNA (gDNA) was extracted from blood samples using a Blood DNA kit (TIANGEN BIOTECH, Beijing, China), and 1 ug of purified gDNA was fragmented into 200–300-base-pair lengths using an ultrasonoscope. Libraries were prepared by performing end-repair, adenylation, and adapter ligation using a protocol provided by the manufacturer of the NGS system (HiSeq2000; Illumina).

A customized capture array (Roche-NimbleGen) containing 307 deafness genes was designed to capture all exons and flanking intron (±10 bp) sequences of the target genes (Supplementary Table 1). The same amount of each library was pooled and then hybridized to the customized capture array. Paired-end reads (PE90) were generated by sequencing using an Illumina HiSeq2000 platform, according to the manufacturer's instructions [28].

### Data filtering, mapping and variant detection

Illumina Pipeline (version 1.9.4) was used to perform image analysis, error estimation and base calling, yielding the primary data. After removing low-quality reads from the primary data using a local algorithm, data analysis and bioinformatics processing were performed based on the reference sequences of the NCBI37/hg19 assembly of the human genome using the Burrows-Wheeler alignment tool. Single nucleotide variations (SNVs) and small insertions and deletions (Indels) were detected using GATK (Genome Analysis Toolkit), and in-house scripts were used for annotation of variants.

### Identification of rare or novel variants

To identify the most likely pathogenic mutations, we filtered out 1) synonymous and non-coding variants (with the exception of splicing site mutations that might create an ectopic splicing site); 2) variants with allele frequencies of 0.0005 or higher in the dbSNP, 1000 Genomes Project, ESP6500 or ExAC database; and 3) missense variants that were predicted to be neutral by the Condel program, which assesses the functional effects of non-synonymous variants using a CONsensus DELeteriousness (Condel) score that combines various tools (SIFT, Polyphen2) [29]. The strength of ectopic splicing sites created by two intronic variants was evaluated by the Human Splicing Finder program (http://www.umd.be/HSF/). The HOPE server (http://www.cmbi.ru.nl/hope/) was used to analyze and predict structural variations in mutant *HOXA1* (homeobox A1) protein and the ConSeq server was used for determining the conservation of *HOXA1* amino acid position 115. Additionally, all rare and novel mutations were checked against the Human Gene Mutation Database (HGMD) to determine whether any identified mutations had been reported to be pathogenic.

### Principle of KEGG pathway enrichment

The number of potential pathogenic genes and all genes in each pathway were determined, and then the number of all potential pathogenic genes and all genes in all pathways were determined separately. Ultimately, a hypergeometric test was applied to determine the statistical significance of the enrichment pathway (*P*), calculated according to the following formula:

$$P=1-\sum_{i=0}^{m-1}\frac{\begin{array}{cc} M & N-M\\ i & n-i \end{array}}{\begin{array}{c} N\\ n \end{array}}$$

where *N* represents the gene count in all pathways, *n* represents the z potential pathogenic gene count in *N*, *M* represents the gene count in each pathway, and *m* represents the potential pathogenic gene count in each pathway. Pathways with a q-value < 0.05, determined from the p-value through multiple comparisons, were considered to be significantly enriched.

### Association analysis of low-frequency variants

To test whether low-frequency mutations (MAF < 0.01 in the 1000 Genomes Project) that might be risk factors for microtia, we performed a gene-based test comparing the burden of low-frequency variants in cases and controls using a sequence kernel association test (SKAT) implemented in the SKAT software package. Only nonsynonymous variants were included in the analysis. Genotype data for the Han Chinese in Beijing (CHB) and Southern Han Chinese (CHS) populations were extracted from the 1000 Genomes Project. Finally, all 32 microtia cases and 208 normal controls from the 1000 Genome Project were genotyped for the selected variants. A Bonferonni correction was used to account for multiple testing (*P* = 2.51 × 10^−4^).

## SUPPLEMENTARY MATERIALS FIGURE AND TABLES



## Abbreviations

NGS: Next generation sequencing
PTA: Pure-tone audiometry
AC: Air conducted
AF: Allele Frequency
KEGG: Kyoto Encyclopedia of Genes and Genomes
