# Supplementary material for: Target sequencing of 307 deafness genes identifies candidate genes implicated in microtia
Source: Oncotarget. 2017 Jun 28;8(38):63324–32. doi: 10.18632/oncotarget.18803 (PMC5609924; doi:10.18632/oncotarget.18803)
Supplement: Supplementary file 1 [file oncotarget-08-63324-s001.pdf]

# Target sequencing of 307 deafness genes identifies candidate genes implicated in microtia

## SUPPLEMENTARY FIGURE AND TABLES

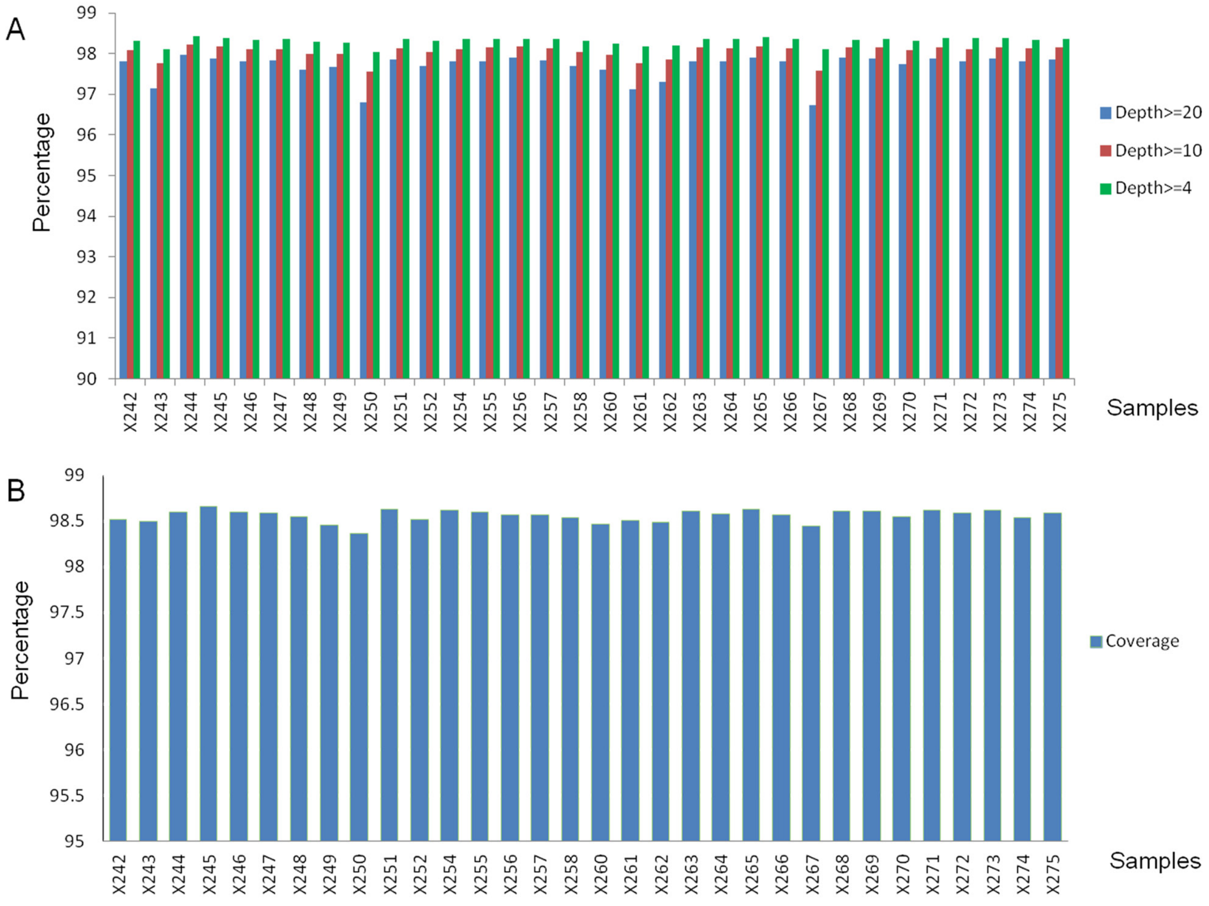


**Supplementary Figure 1: Coverage analysis of NGS sequencing of 307 targeted genes in 32 individuals. (A)** Percentage of bases at the different read depths. **(B)** Percentage of fraction of the targeted bases covered for each individual.

**Supplementary Table 1: Summary of the 307 targeted genes.**

**See Supplementary File 1**

**Supplementary Table 2: Results of gene-based, low-frequency variant association tests (significance level P = 2.51 × 10-4).**

**See Supplementary File 2**
